# Reconfigurable Shape Memory and Self-Welding Properties of Epoxy Phenolic Novolac/Cashew Nut Shell Liquid Composites Reinforced with Carbon Nanotubes

**DOI:** 10.3390/polym10050482

**Published:** 2018-04-28

**Authors:** Pornnapa Kasemsiri, Narubeth Lorwanishpaisarn, Uraiwan Pongsa, Shinji Ando

**Affiliations:** 1 Department of Chemical Engineering, Khon Kaen University, Khon Kaen 40002, Thailand; l.narubeth@gmail.com; 2 Division of Industrial Engineering Technology, Rajamangala University of Technology Rattanakosin, Wang Klai Kang, Won Campus, Prachuap Khiri Khan 77110, Thailand; uraiwan.pon@rmutr.ac.th; 3 Department of Chemical Science and Engineering, Tokyo Institute of Technology, Ookayama 2-12-1-E4-5, Meguro-ku, Tokyo 152-8552, Japan

**Keywords:** shape memory, stress relaxation, welding/joining, thermal properties

## Abstract

Conventional shape memory polymers (SMPs) can memorize their permanent shapes. However, these SMPs cannot reconfigure their original shape to obtain a desirable geometry owing to permanent chemically or physically crosslinked networks. To overcome this limitation, novel SMPs that can be reconfigured via bond exchange reactions (BERs) have been developed. In this study, polymer composites consisting of epoxy phenolic novolac (EPN) and bio-based cashew nut shell liquid (CNSL) reinforced by multi-walled carbon nanotubes (CNTs) were prepared. The obtained composites exhibited shape memory and self-welding properties, and their shapes could be reconfigured via BERs. Their shape memory mechanisms were investigated using variable-temperature Fourier transform infrared spectroscopy and dynamic mechanical analysis. The EPN/CNSL composite containing 0.3 wt % CNTs showed the highest shape fixity and shape recovery ratio. Furthermore, shape memory behavior induced by irradiation of near-infrared (NIR) light was also observed. All samples showed high shape recovery ratios of nearly 100% over five cycles, and increasing the CNT content shortened the recovery time remarkably. The ability of shape reconfiguration and stress relaxation affected the photo-induced shape memory properties of reshaped samples. Additionally, the self-welding properties were also influenced by stress relaxation. The hindrance of stress relaxation caused by the CNTs resulted in a decrease in adhesive fracture energy (*G*_c_). However, the *G*_c_ values of EPN/CNSL composites were comparable to those of epoxy vitrimers. These results revealed that the material design concepts of thermal- and photo-induced shape memory, shape reconfiguration, and self-welding were combined in the EPN/CNSL composites, which could be feasible method for advanced smart material applications.

## 1. Introduction

Shape memory polymers (SMPs) are a type of smart material that have been widely used in applications, such as aerospace structures, sensors, textiles, and self-crack-healing applications. SMPs can recover from deformed temporary shapes to their original shapes in response to external stimulus, such as heat, moisture, pH, and electric or magnetic fields [1]. Recently, new types of SMPs have been developed towards the production of multi-stimuli-responsive polymers for various applications, such as photo-actuator, photo-sensor, and photo-thermal image [2]. Photo-thermal fillers, such multi-walled carbon nanotubes (CNTs) have been widely used as reinforcing agents for SMP matrices, owing to their excellent thermal, mechanical, and optical properties [3]. CNTs were successfully incorporated into photo-actuator based thermoplastic and thermosetting [4,5]. Yi et al. [6] reported an SMP containing CNTs and graphene oxide that was photothermally activated by near-infrared (NIR) laser. CNTs can efficiently absorb NIR radiation and convert it to thermal energy to enhance the shape memory behavior of an SMP. Li et al. [7] reported that a reinforcement of an epoxy with CNTs increased the deformation temperature of its shape memory behavior and the temperature depended on the load transfer efficiency at the interface between the CNTs and epoxy matrix. To achieve high performance CNTs composite, well dispersion is a key parameter. Generally, CNTs are agglomerated by strong van der Waals interactions. Various techniques, such as shearing, sonication, and functionalization, have been applied to overcome the agglomeration of CNTs in polymer matrix [8,9].

The complex geometry of reconfigurable SMPs can currently be simplified through bond exchange reactions (BERs). Leiber and coworkers [10] recently purposed a new type of polymer known as ”vitrimers” that contain dynamic and exchangeable covalent bonds. The network of a vitrimer can exchange its covalent bonds at a temperature higher than the glass transition temperature (*T*_g_). A thermally induced BER can release the internal stress of polymer networks and allow the materials to be reshaped, welded, and reprocessed. In general, the degree of stress relaxation of a vitrimer can be estimated through temperature and time, at which the viscosity was reduced to 10^12^ Pa∙s or the stress relaxation modulus was lowered to 37% of an initial value [11,12]. According to previous studies, the stress relaxation behaviors of vitrimer depends on several factors: (i) the number of reactive functional groups capable of BERs; (ii) the occurrence of other reactions that consume the reactive functional groups capable of BER, but are incapable of bond exchange; and (iii) the amount of catalyst for the acceleration of the BERs [13,14]. The use of catalysts, such as Zn(II) and imidazole, are necessary for the preparation of epoxy vitrimers [15,16]. Stress relaxation to a target value leads to the high performance of vitrimers. Altuna et al. [16] investigated an epoxy vitrimer cured with carboxylic acids and imidazole as a catalyst. The characteristic properties of reconfigurable shapes of the epoxy vitrimer were observed at 160 °C for 1 h via BERs of esterification and transesterification. The shape fixities and the shape recovery ratios of the samples were ca. 99%. In the case of partial stress relaxation, the characteristics of vitrimers can be partly observed. Imbernon et al. [14] reported self-adhesion of epoxidized natural rubber (ENR) via peroxide crosslinks. The stress relaxations of the samples decreased slightly to 55–98% of the initial values. Self-adhesiveness and low detachment energy were observed at lower crosslinking densities. 

The development of epoxy composites that exhibit shape memory properties has been extensive, owing to their high moduli, high-temperature performances, and high shape fixities and shape recovery ratios [17]. Recently, the development of epoxy-based renewable organic materials has also attracted much attention from academia and industry. Most of bio-based epoxy resins have been focused on the synthesis of epoxy monomers from vegetable oil [18] and phenolic compounds found in plants [19]. Interestingly, various types of bio-based organic compounds, such as lignin [20], castor oil, and cashew nut shell liquid (CNSL) [21], can be used directly as hardeners in the curing process of epoxy resins. The present authors [22] have reported that an epoxy cured with naturally-derived CNSL shows good adhesion properties comparable with those of epoxies cured with commercial hardeners. Epoxy/CNSL featured two types of crosslinking network generated via etherification and esterification. The interpenetrating network of epoxy/CNSL has high potential to provide shape memory properties. Furthermore, many ester linkages in epoxy/CNSL can be interchanged through transesterification, which can release internal stress to afford reconfigurable shape memory and self-welding properties. 

To our best knowledge, there have been no reports on the shape memory properties of composites consisting of epoxy phenolic novolac (EPN) and bio-based CNSL as a hardener obtained from renewable organic materials. Therefore, the objectives of this study were to develop novel smart materials composed of epoxy/CNSL composites reinforced with CNT and to investigate their curing process, stress relaxation, shape reconfiguration and self-welding properties. 

## 2. Experimental Section

### 2.1. Materials

**Scheme 1:**
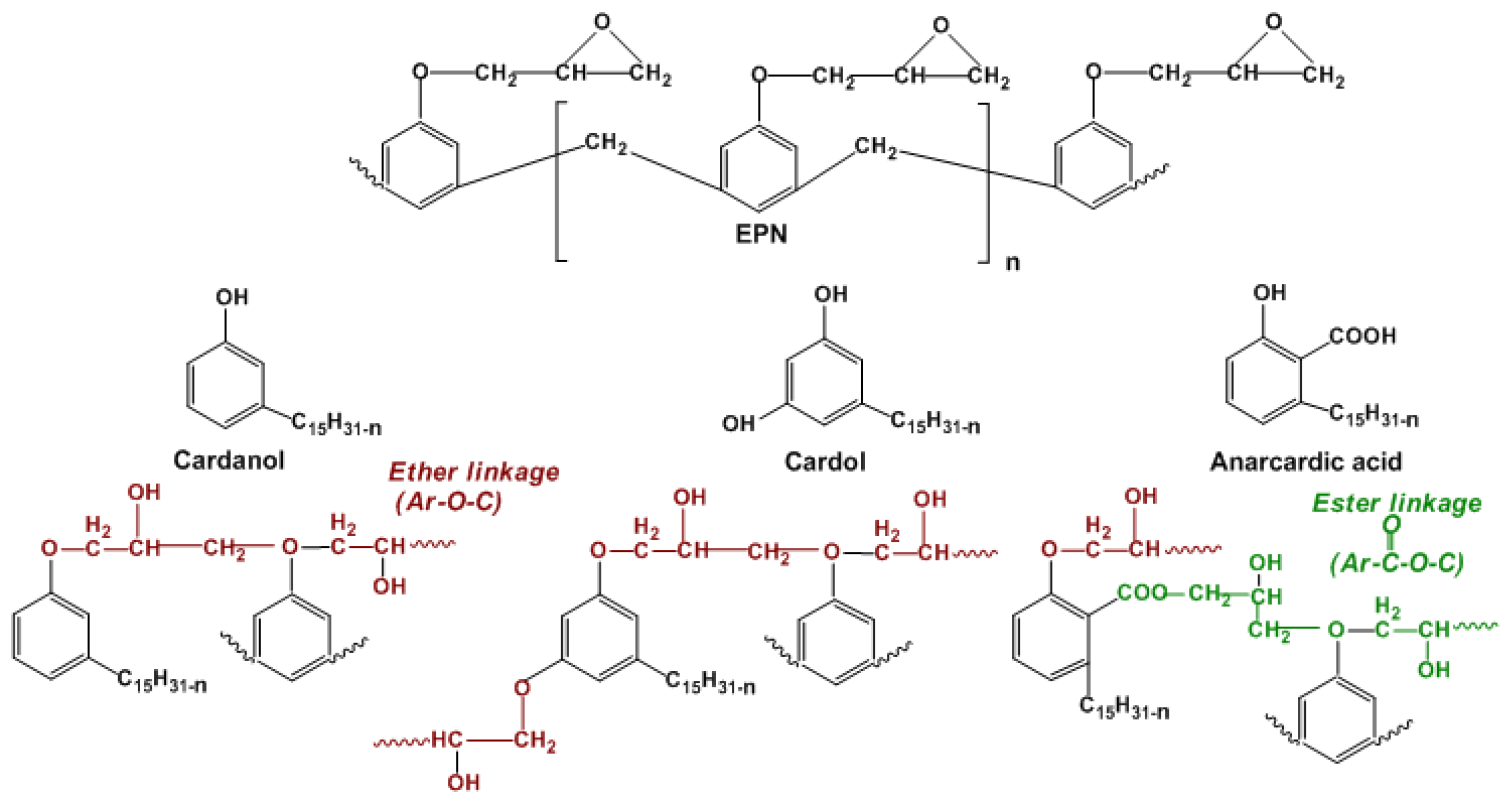
Possible curing reactions between epoxy phenolic novolac (EPN) and cashew nut shell liquid (CNSL).

EPN (grade YDPN 631) was obtained from Aditya Birla Chemicals, Thailand. Naturally-derived CNSL was supplied by Mahboonkrong Sirichai 25 Ltd., Nakhon Ratchasima, Thailand. CNSL consisted of cardol (36.2%), cardanol (31.5%), anacardic acid (24.6%), and traces of methyl cardol, as shown in Scheme 1. Multi-walled CNTs produced using an infusion chemical vapor deposition process were obtained from Nanogen, Thailand. The length and outer diameter of the CNTs were 3–12 μm and 12.9 nm, respectively. An image of CNTs is shown in Supplementary Materials Figure S1.

Composite samples were prepared by mixing EPN/CNSL matrix at 70:30 *w/w* with 0−0.5 wt % CNTs, as shown in Supplementary Materials Figure S2. First, EPN was dissolved in dimethylformamide using a planetary mixing machine (ARE-300, Thinky Corp., Tokyo, Japan) at 2000 rpm for 7 min, and then CNTs were dispersed in the mixture solution using an ultrasonic homogenizer (US-50, Nihonseiki Kaisha Ltd, Tokyo, Japan) for 15 min in an ice bath, and CNSL was added. Subsequently, the mixture solution was stirred for another 30 min to obtain homogenous solution and then casted on a glass plate. The sample was cured stepwise at 85, 140, and 170 °C for 1 h at each temperature and 200 °C for 2 h under a nitrogen atmosphere. After the thermal curing process, exothermic heat was not detected indicating that the samples were fully cured, as shown in Supplementary Materials Figure S3.

### 2.2. Measurements

#### 2.2.1. Evaluation of Curing Reaction

The thermal curing behaviors of the composites were monitored using a differential scanning calorimeter (DSC-60, Shimadzu, Kyoto, Japan). The heating rate was 10 °C/min from 30 to 250 °C under a nitrogen gas purge. The structural changes of EPN/CNSL during the curing process were examined using in situ FT-IR (FT-IR, JASCO 4200 Crop., Tokyo, Japan) with an instrument equipped with a hot stage (10002L, Linkam Crop., Tadworth, UK). 

The variable-temperature FT-IR measurements were carried out from 30 to 250 °C at a heating rate of 3 min/°C under a nitrogen flow. The absorbance of selected peaks was normalized using the C=C stretching vibration of the benzene rings at 1507 cm^−1^ as internal standard, to compensate for the variation of the thickness during curing. All of the samples were sufficiently thin to obey the Beer-Lambert law. The degree of conversion was estimated using the peak intensities of reactive groups assignable through FT-IR as Equation (1):

(1) $$\alpha =1-\frac{A_{T,i}}{A_{T,0}}$$

where α is the conversion, and *A*_T,0_ and *A*_T,i_ are the absorbance of reactive group before curing and at each temperature *T*, respectively. 

#### 2.2.2. Analysis of Thermomechanical Properties

**Scheme 2:**
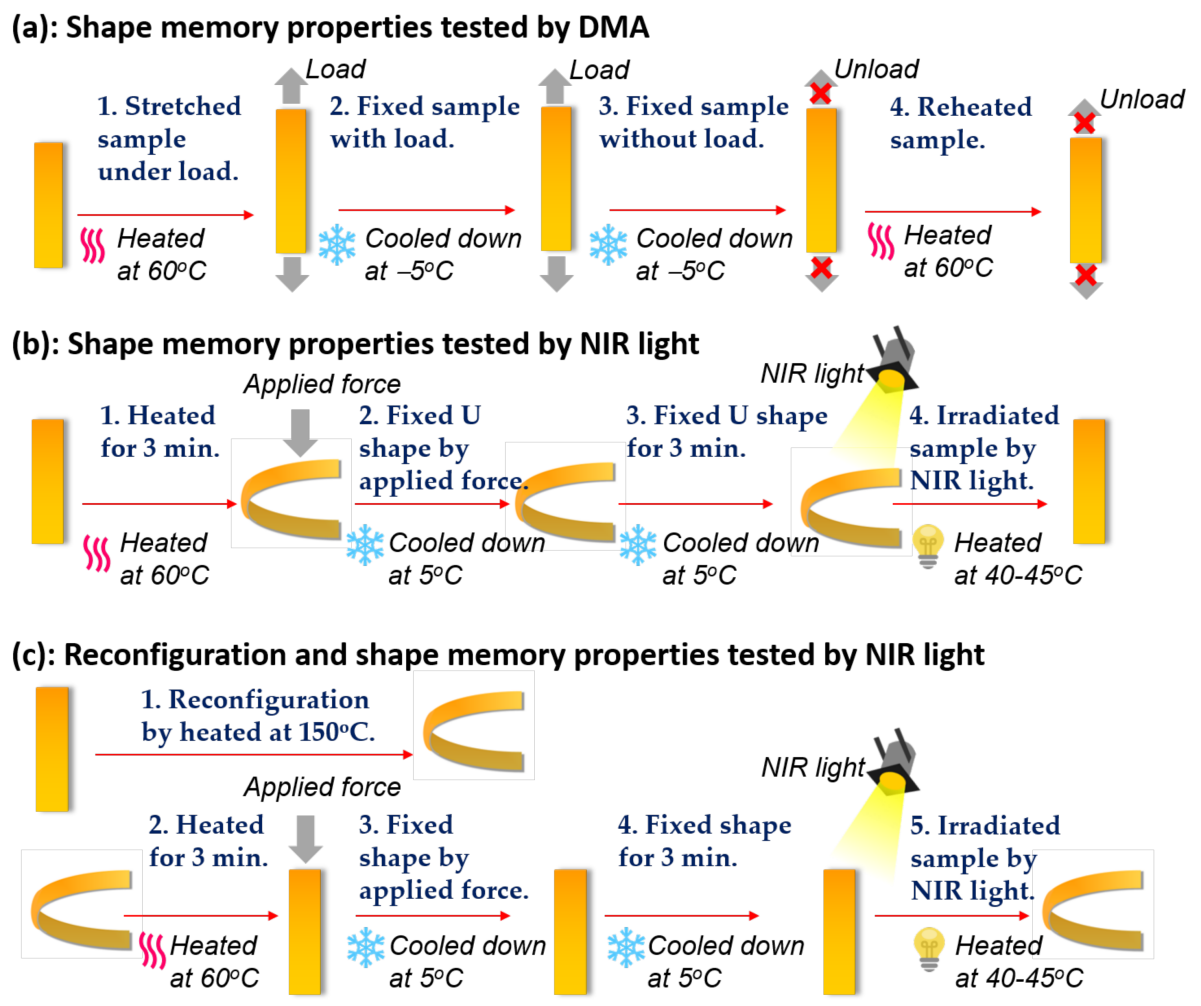
Examination of shape memory properties using DMA and NIR light.

The dynamic mechanical properties and relaxation behaviors of the composites were examined using dynamic mechanical analysis (DMA, Hitachi 7100, Tokyo, Japan). The temperature sweep experiment was conducted using a frequency of 1 Hz and a strain value of 0.1% in tensile mode. The temperature was scanned at 3 °C/min under a nitrogen atmosphere. To evaluate the shape recovery ratios and shape fixities of the composites, thermomechanical cycle tests were conducted through DMA in tensile mode [23]. The experimental procedure is shown in Scheme 2a. The specimens were stretched to the certain strain (ε_m_) under a constant force at 60 °C (step 1). The strain of the specimen was maintained, and the temperature was lowered to −5 °C (step 2). The strain of the specimen (ε_u_) was measured after unloading at −5 °C (step 3). The degree of shape fixity (*R*_f_) was calculated using Equation (2).

(2) $$R_{f}=\frac{\varepsilon_{u}}{\varepsilon_{m}}$$

To evaluate the shape recovery capacity, the specimens were heated from 0 to 60 °C without load, and held for 10 min (step 4), and then the recovered strains of the specimens (ε_p_) were measured. The shape recovery ratio (*R*_r_) was calculated using Equation (3).

(3) $$R_{r}=\frac{\varepsilon_{m}-\varepsilon_{p}}{\varepsilon_{m}}$$

The heating and cooling rates were 5 °C/min, and the loading and unloading rates of tensile stress were 0.05 N/min. 

#### 2.2.3. Shape Memory Phenomena Induced by NIR

A 150 W tungsten halogen lamp (Philips 7158, 24 V, Philips Lighting, Amsterdam, Netherland) in a dedicated light house (JASCO, Tokyo, Japan) was used as the source of NIR radiation. The distance between the light source and film samples was 5 cm. The light intensity at the sample position was measured to be 12 mW/cm^2^ using a photo intensity meter (Advantest Q8221, Tokyo, Japan). The dimensions of the samples were 30 mm × 10 mm, and the thickness was ca. 400 μm. The experimental procedure is shown in Scheme 2b. First, samples were deformed into U shapes at 60 °C and then cooled at 5 °C for 3 min to fix the deformation. Subsequently, the deformation was recovered by exposing the samples to the light. The shape recovery times were determined by using a digital camera, and the shape recovery ratios were calculated as shown in Supplementary Materials Figure S4. 

#### 2.2.4. Stress Relaxation Behavior

The stress relaxation behaviors of the samples were evaluated using another DMA (TA instruments Q800, New Castle, DE, USA) at 130, 140, 150, and 160 °C. The samples were equilibrated at each temperature for 10 min before being subjected to an instantaneous strain of 1%. The modulus relaxation was monitored as a function of time. 

#### 2.2.5. Self-Welding Properties

A T-peeling test based on the standard of the ASTM D1876 (American Society for Testing and Materials, West Conshohocken, PA, USA) was adopted to measure the interfacial fracture energy of the self-welding samples [24]. Two specimens with identical dimensions of 30 mm × 10 mm × 0.40 mm were welded together using a pressure of 0.15 g/cm^2^ at 150 °C for 1 h. An open region was left at the end of the welded samples. The stress-strain behaviors of the welded samples were tested based on the adhesive fracture energy of peeling using DMA (Hitachi 7100, Tokyo, Japan) in tensile mode at 25 °C. The grips were separated at a constant rate of approximately 0.5 mm/min, as shown in Supplementary Materials Figure S5.

#### 2.2.6. Scanning Electron Microscopy (SEM) Observation

The microscopic analyses of the fractured epoxy surfaces and composite samples were conducted using field emission scanning electron microscopy (FE-SEM, Hitachi S4700, Tokyo, Japan). The acceleration voltage was 10 kV. The samples were free fractured in liquid nitrogen, and then sputtered with osmium tetroxide 2 nm using an osmium coater (Neoc Pro, Meiwafocis, Tokyo, Japan).

## 3. Results and Discussion

### 3.1. Curing Reaction of EPN/CNSL Composites

**Figure 1:**
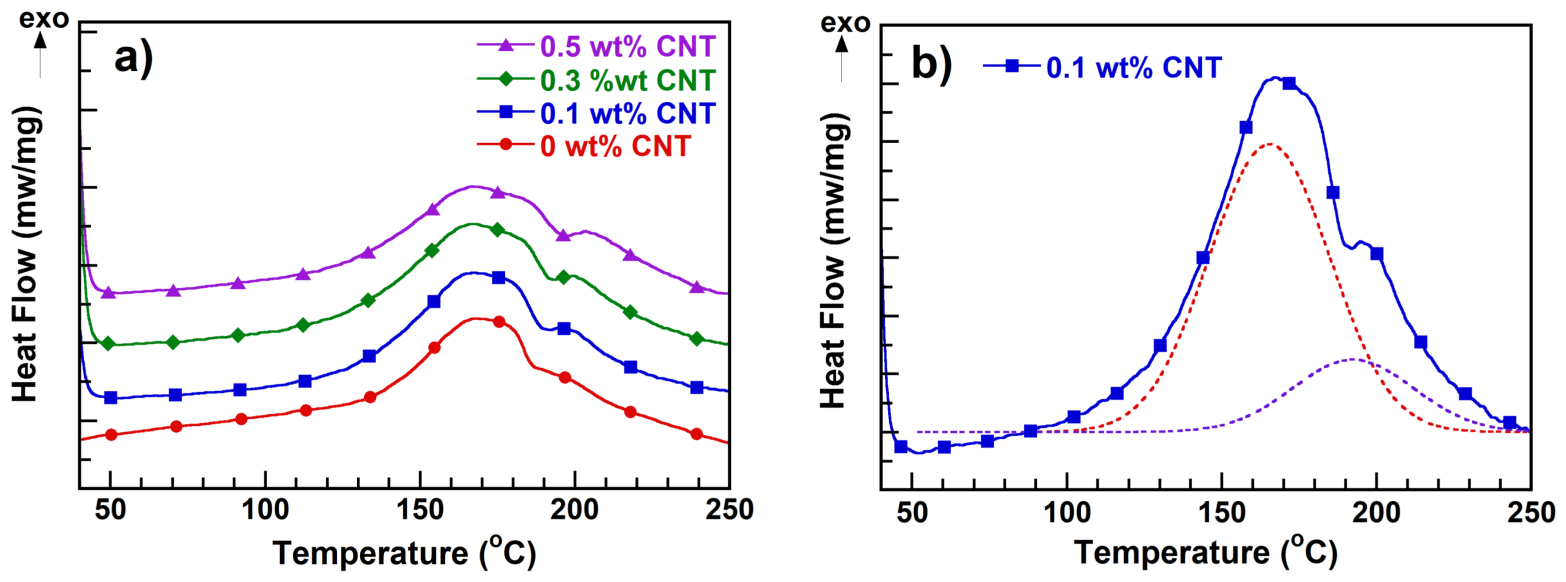
(**a**) DSC thermograms of EPN/CNSL containing 0–0.5 wt % CNT and (**b**) separated peaks of EPN/CNSL containing 0.1 wt % CNTs.

The DSC charts for the curing reactions of EPN/CNSL at 0−0.5 wt % CNTs are shown in Figure 1a. As shown in Figure 1b, each sample showed two overlapping peaks, indicating the existence of two curing stages. The overlapping peaks were separated using Origin 8.1 software, and the results are summarized in Supplementary Materials Table S1. The first exothermic peak is attributable to the reaction between epoxide rings and the major components of CNSL i.e., cardanol, cardol, and anacardic acid. The second exothermic peak is attributable to the esterification between carboxylic groups and epoxide rings generated in the first curing reaction [22], as depicted in Scheme 1. The first and second curing reactions occurred in the 166−168 °C and 189−203 °C temperature ranges, respectively. It is clear that the increase of CNT content causes the second curing reaction to occur at higher temperatures, owing to the “hindrance effect” of CNTs on the mobility of the polymer chain. Puglia et al. [25] reported that entangled CNT structures increase the viscosity of epoxy, leading to decreased segment mobility during vitrification. Yang et al. [26] reported that CNTs decelerate the propagation reactions and extend the gelation time of epoxy. Xie et al. [27] also reported that CNTs hinder the propagation reaction of epoxy at high temperatures owing to decreased in segment mobility.

**Figure 2:**
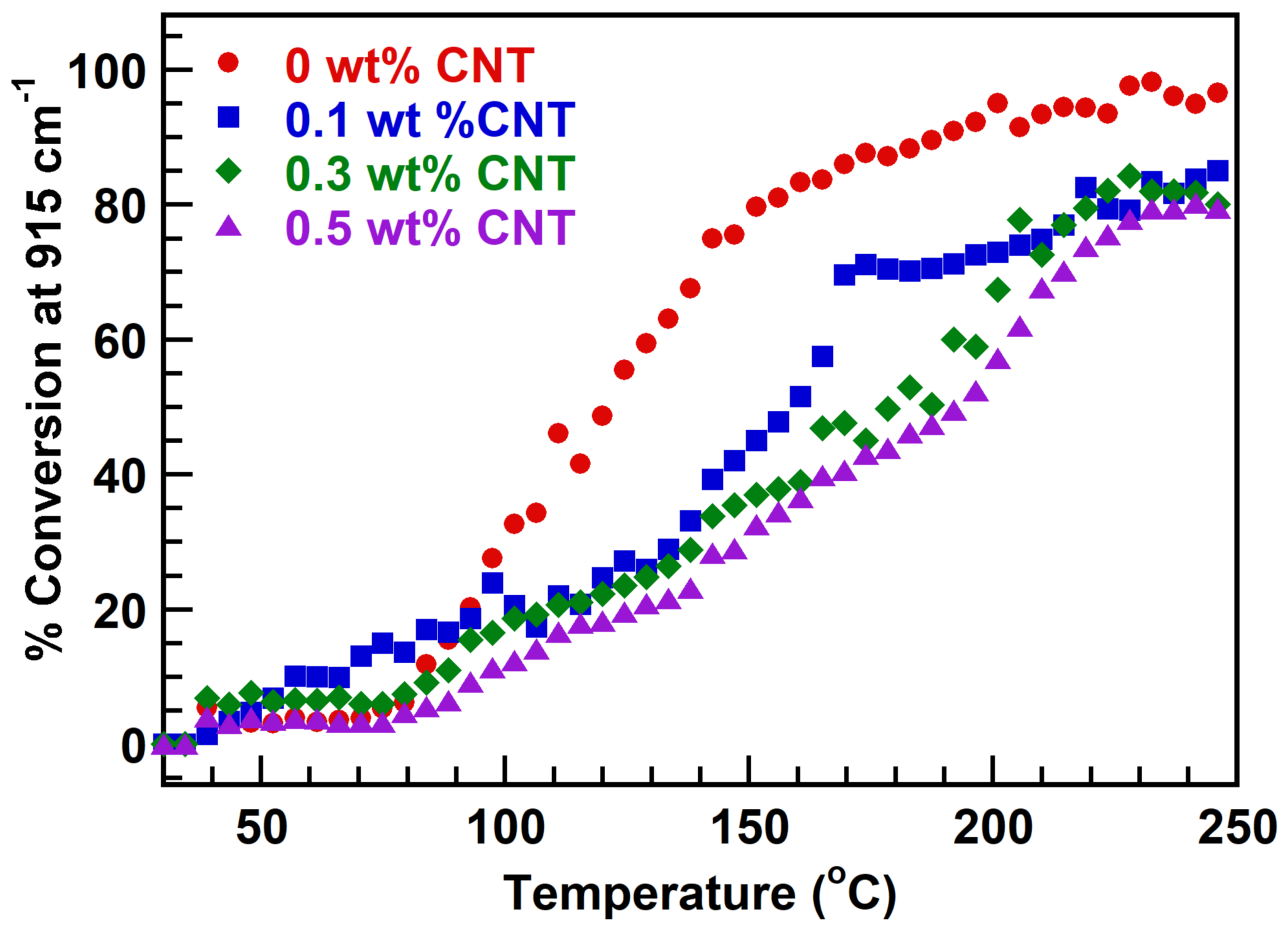
Temperature dependence of the degrees of conversion determined using variable temperature in situ FT-IR spectroscopy.

The curing mechanism for EPN/CNSL containing CNTs was investigated using in situ FT-IR measurements. The FT-IR spectra of EPN monomer, CNSL, and EPN/CNSL film composites are shown in Supplementary Materials Figure S6, and the wavenumbers and signal assignments are summarized in Supplementary Materials Table S2. EPN monomer shows a major characteristic peak of epoxide rings at 915 cm^−1^. The absorption bands at 2864 and 1453 cm^−1^ can be assigned to the C−H and C−CH_2_ stretching vibrations of epoxide rings [28]. The absorption bands of CNSL appeared at 1007 and 912 cm^−1^, which are attributable to the phenolic group at the meta position. The characteristic C=O stretching bands of anacardic acid appeared at 1650 cm^−1^ [22,29]. After the curing process, the intensity of the peak at 915 cm^−1^ decreased substantially, indicating the opening of the epoxide rings followed by crosslinking [30]. Furthermore, two new peaks were detected at 1130 and 1750 cm^−1^ for all of the samples, as shown in Figure 2a,b. The former corresponds to the stretching of C−O−C bonds generated in the reaction between the epoxy and the hydroxyl groups of cardanol and cardol, and the latter can be assigned to the C=O stretching following the reaction between the epoxy and carboxylic acid [31]. The peak at 1750 cm^−1^ can be detected at 180−250 °C, as shown in Supplementary Materials Figure S6, which coincides well with the curing temperature of the second reaction. These chemical reactions are shown in Scheme 1. It should be noted that, at a higher amount of epoxy groups than polycarboxylic acids, the −OH groups derived from the reacted epoxy were thus reacted with the epoxide groups at a slow reaction rate, to perform polyetherification with a high conversion of curing [32].The absorption at 915 cm^−1^, characteristic of epoxide rings, was used as an index for the ring-opening reaction. The degrees of conversion estimated from this peak at elevated temperatures are given in Figure 2. The degrees of conversion were 80−98% at 250 °C, and decreased with the increasing CNT content. This is also because of the hindrance effect of CNT particles, which lower the mobility of the epoxy monomers.

### 3.2. Thermomechanical Properties of EPN/CNSL Film Composites

**Figure 3:**
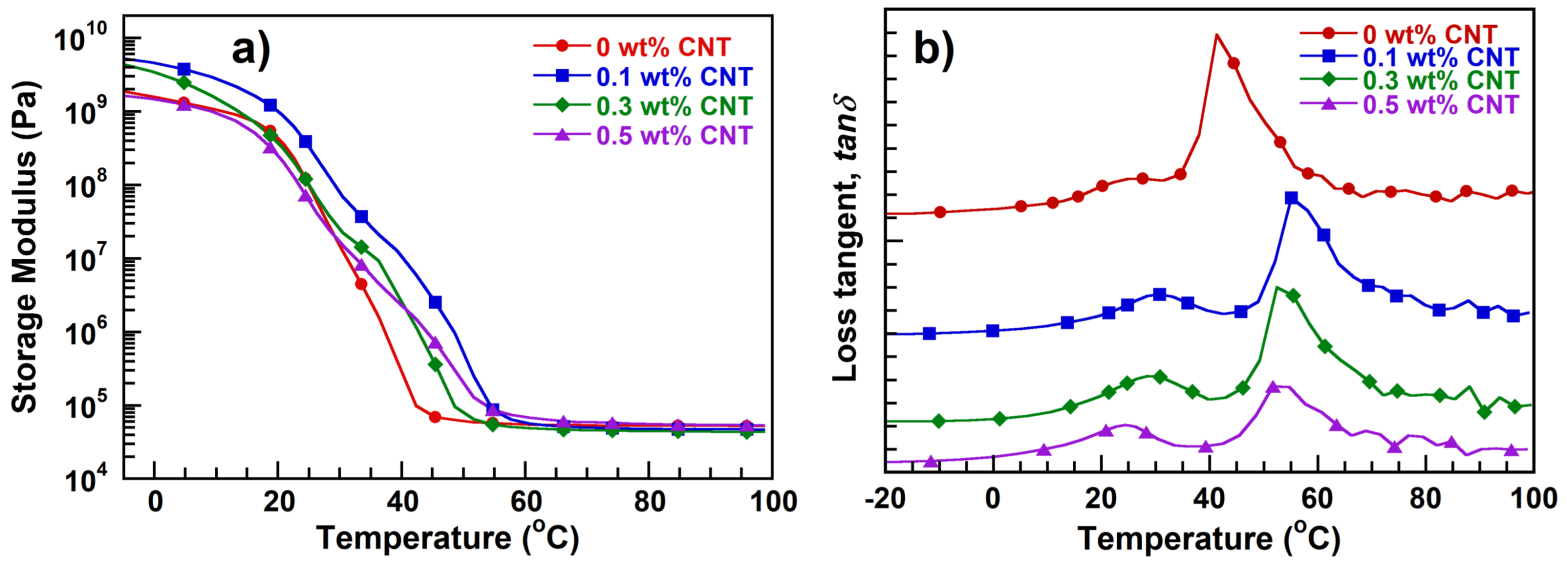
(**a**) Storage modulus and (**b**) tanδ curves of EPN/CNSL composites.

**Figure 4:**
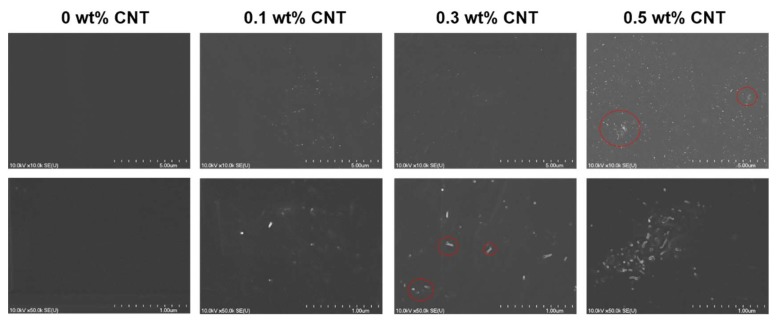
Scanning electron micrograph of EPN/CNSL composites with different CNT contents.

The temperature dependences of the storage moduli (*E*’) and tanδ of the composite samples are presented in Figure 3a,b, respectively. The storage modulus of EPN/CNSL matrix in the glassy state was 1.54−4.77 GPa, and it increased remarkably when 0.1 wt % CNTs were incorporated. This can be explained through the reinforcing effect of the homogeneously dispersed CNT nanofiller. Figure 4 shows the SEM images of EPN/CNSL containing 0−0.5 wt % CNTs. The EPN/CNSL matrix had a smooth fracture surface, and homogenous dispersion of CNTs was observed in the composite with 0.1 wt % CNTs. An increase in CNT content to 0.3 wt % led to some aggregation, and the formation of CNT clusters was observed at 0.5 wt %. The poor dispersion of CNTs at higher contents in the epoxy matrix is the result of fewer interactions between the inherently inert/hydrophobic CNTs and the polar/hydrophilic epoxy, as well as the attraction between the CNTs via van der Waals interactions [33,34].

The tanδ value of a sample is defined as the loss ratio of storage modulus. The *T*_g_ was determined from the peak of tanδ, as shown in Figure 3b. All samples showed a shoulder peak at 24.8−26.2 °C and a main peak at 41.6−55.4 °C. These characteristic peaks can be attributed to the interpenetrating network (IPN) consisting of the ether and ester linkages of EPN/CNSL. Tan et al. [35] reported that 50/50 composites of epoxidized soy bean oil/diglycidyl ether of bisphenol A showed two tanδ peaks, which were respectively related to etherification and esterification. In general, IPN structures are formed during curing processes, and the lack of polymer network compatibility induces thermodynamically-driven phase separation. Therefore, an IPN possesses two mechanical relaxations of domains [36]. At the β-relaxation process at 24.8−26.2 °C, a sharp decrease in *E*’ value was observed for all samples, which indicated a character of glass-rubber transition in soft segments. EPN/CNSL containing 0.1 wt % CNTs showed the highest *T*_g_ (~55 °C). The compatibility between the CNTs and the polymer matrix resulted in homogeneous dispersion of the CNTs, which restricts the mobility of the polymer chains, and thus, increases the *T*_g_ [37]. 

### 3.3. Shape Memory Effect of EPN/CNSL Composites

**Figure 5:**
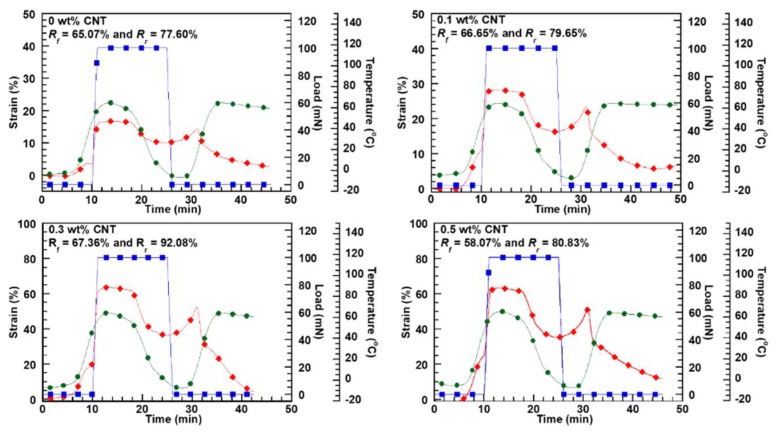
Shape memory properties of EPN/CNSL composites tested by DMA: (■) load, (♦) strain, and (●) temperature.

**Figure 6:**
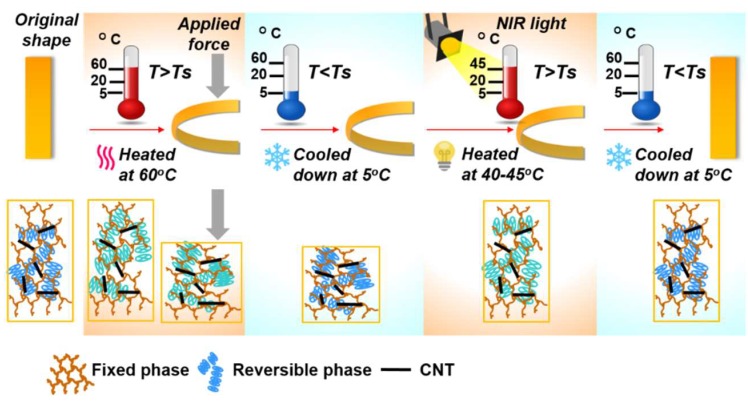
Shape memory behavior EPN/CNSL composites during NIR light test.

The stress-strain-temperature-time diagrams for the shape memory properties were obtained using DMA and are shown in Figure 5. The programmed procedure was performed through the uniaxial stretching of a sample at a temperature above the *T*_g_, followed by rapid cooling to −5 °C at constant load. Afterwards, the load was released, and the samples were reheated to 60 °C. Shrinkage was observed for all the samples upon the removal of load, owing to the low *T*_g_ of the soft segment. This behavior is consistent with that of polyurethane SMP reinforced with CNTs [38]. Increasing the CNT content up to 0.3 wt % significantly enhanced the shape memory properties. In general, SMPs consist of two major structures, the hard and soft segments, which act as the fixed phase and reversible phase, respectively, as illustrated in Figure 6. When stress is applied, the fixed phase prevents free motion and displacement of surrounding polymer chains, whereas the reversible phase is deformed during shape memory cycle. These phases act as a molecular switch to fix and recover the deformed shape at above and below the switching temperature (*T*_s_), respectively [39]. Gu et al. [40] observed that the interaction between the CNTs and the fixed phase of the SMP improved the shape fixity. Furthermore, the presence of CNTs in an SMP can store elastic stain energy and helps strain recovery during stretching and recovering processes. As shown in Figure 5, the addition of 0.5 wt % CNTs resulted in slight decreases in the shape fixity and the shape recovery ratio. The formation of CNT clusters weakened the interfacial adhesion between the CNTs and the polymer matrix, which led to the failure of load transfer between the two phases [41]. Ni et al. [42] reported that the addition of excess filler results in a reduction in the shape memory effect owing to the self-aggregation of the filler. 

### 3.4. Shape Memory Behavior of EPN/CNSL Composites Induced by NIR Irradiation

The shape recovery images of EPN/CNSL containing 0–0.5 wt % CNT are shown in Supplementary Materials Table S3, and the recovery times are summarized in Table 1. All samples showed 100% shape recovery after different irradiation periods. When the samples were irradiated with NIR light, the light was absorbed efficiently by the CNTs converted to thermal energy and transferred to the polymer matrix, which increases the temperature of the entire sample. At temperatures above the *T*_s_, the reversible phase of an SMP possesses high elasticity owing to the flexibility of the chain segment, which allows it to take a temporary shape. At temperatures below the *T*_s_, this shape is automatically fixed because of the limited flexibility of the chains. When the SMP was heated again above the *T*_s_, it spontaneously recovered to its original shape; the conformational entropy of the molecular segments was the driving force. During the heating process, the molecular mobility increased, leading to an increase in conformational entropy [6,43], as illustrated in Figure 6. The shape recovery times of the samples decreased with increasing CNT content. Faster recovery indicates that the CNTs enhanced the light absorption and thermal energy transfer of the SMP composites. EPN/CNSL containing 0.5 wt % CNT showed the shortest recovery time. A small amount of aggregated CNT enhanced the thermal conductivity by decreasing the thermal contact resistance among the tubes [44]. Furthermore, each sample displayed consistent shape recovery rates after five testing cycles, which indicates that the shape recovery effect is highly stable, as shown in Table 1. Slight reductions in the shape recovery ratios and recovery times were observed for all the samples, possibly because the molecular materials are likely to fatigue during repeated shape memory processes [45].

### 3.5. Stress Relaxation of EPN/CNSL Composites

The stress relaxation behaviors of the EPN/CNSL composites examined at 130−160 °C are shown in Supplementary Materials, Figure S7. The relaxation rate increased with increasing temperature. It should be noted that stress relaxation occurred in our composites in the absence of a catalyst. The normalized stress relaxations (*E*/*E*_0_) of all samples decreased to 0.75 as a function of time. It should be noted that the final *E*/*E*_0_ values were significantly larger than those of the vitrimers that require a catalyst (*E*/*E*_0_ = 0.37) [46,47]. The similar relaxation rates of EPN/CNSL composites at 150 and 160 °C were observed. Therefore, the stress relaxation at 150 °C at 1 h was used to perform the reconfiguration of shape and self-welding. The stress relaxation resulted from the transesterification BER and the rearrangement of the flexible network. Therefore, the degree of BER was related to the amount of ester linkages in system. The consumption of free hydroxyl (−OH) groups of EPN/CNSL by etherification and esterification might be an explanation for the relatively low release of stress. In this case, the etherification is a non-reversible reaction that decreases the number of ester linkages. Furthermore, the amounts of ester linkages in EPN/CNSL composites were limited by the low content of anarcardic acid (24.7%) comparable with those of the other components in CNSL. The quantitative estimation of the degree of BER in EPN/CNSL composites should be further studied to clarify and consolidate the character of the material. The degree of transesterification BER in epoxy acid networks was estimated by using gas chromatography-mass spectrometry [47]. Imbernon et al. [14] reported a similar observation; the stress relaxation of ENR cured with peroxide was found to be 0.8 of the initial value, because a side reaction hindered the reversible reaction of the disulfide bonds. 

**Figure 7:**
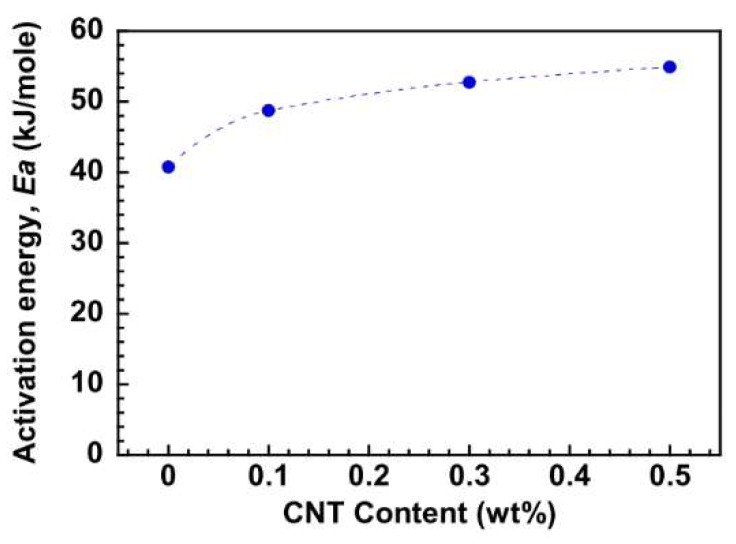
Activation energy for stress relaxation of EPN/CNSL composite.

In the stress relaxation experiment, the characteristic relaxation time was defined as the time required for a particular stress relaxation modulus (τ). For the EPN/CNSL composites, the time, τ, at which the *E*/*E*_0_ value becomes 0.75 was plotted against the inverse temperature, and the activation energy was estimated according to the Arrhenius equation [48], (Equation (4)).

(4) $$\ln\tau =\frac{E_{a}}{RT}-\ln A,$$

where *E*_a_ is the activation energy of stress relaxation (kJ/mol) caused by transesterification, *R* is the universal gas constant, and *T* is the testing temperature (K). The values of *E*_a_ shown in Figure 7 are in the 40.73−54.91 kJ/mol range, and increased slightly with increasing CNT content. This can be explained by the fact that the incorporation of CNTs hinders the stress relaxation of the polymer matrix by restricting the molecular motion of the polymer chains at the interface [13].

### 3.6. Reconfiguration and Shape Memory of EPN/CNSL Composites

Stress relaxation behavior is an important factor in the production of materials that exhibit shape reconfigurability and shape memory properties [49]. In order to determine how to combine these two properties, samples were heated at 150 °C for 1 h to obtain new permanent shapes. The reconfigurability of the samples was tested by reheating them at 150 °C. It was clear that the EPN/CNSL matrix exhibited a better ability to fix the reconfigured shape compared to the other composites as shown in Table 2. The newly configured shapes of the EPN/CNSL composites (U shape) were not fully maintained after heating, and partial recovery to the original shapes was observed when the CNT content was 0.1−0.5 wt %. This result implies that the composites containing CNTs cannot relax the internal stress sufficiently to afford a good permanent shape. Brutman et al. [11] suggested that complete of stress relaxation via BERs lowers the stress and strain of an SMP to make its new shape permanent after heating. Shape reconfiguration would be a new alternative method for processing products with complicated geometries without using plastic molding. The shape memory performance after shape reconfiguration was also studied using the experimental procedure shown in Figure 2c. After the samples were reshaped, the shape memory procedure was performed several times from a new permanent shape created using BERs, as illustrated in Table 2. The shape recovery ratios and recovery times of the reshaped samples are summarized in Table 3. The EPN/CNSL matrix showed the highest shape recovery ratio, which was in the 85.3−97.9% range after five test cycles. The shape recovery ratio decreased with increasing CNT content, which could be attributed to the low degree of stress relaxation and the shape recovery effect after reconfiguration. Ma et al. [50] observed that the recovery ratio of a bio-based epoxy containing disulfide bonds depends on the degree of stress relaxation. The recovery times of the EPN/CNSL composites decreased with increasing CNT content. The incorporation of 0.5 wt % CNTs effectively shortened the recovery time by ca. 90%. As mentioned above, this result is consistent with the recovery times of EPN/CNSL composites with CNTs. The variations in shape recovery ratios and shape recovery times observed in the reconfigurable EPN/CNSL composites could be applied for the design of sophisticated shape memory systems. 

### 3.7. Self-Welding of EPN/CNSL Composites

**Figure 8:**
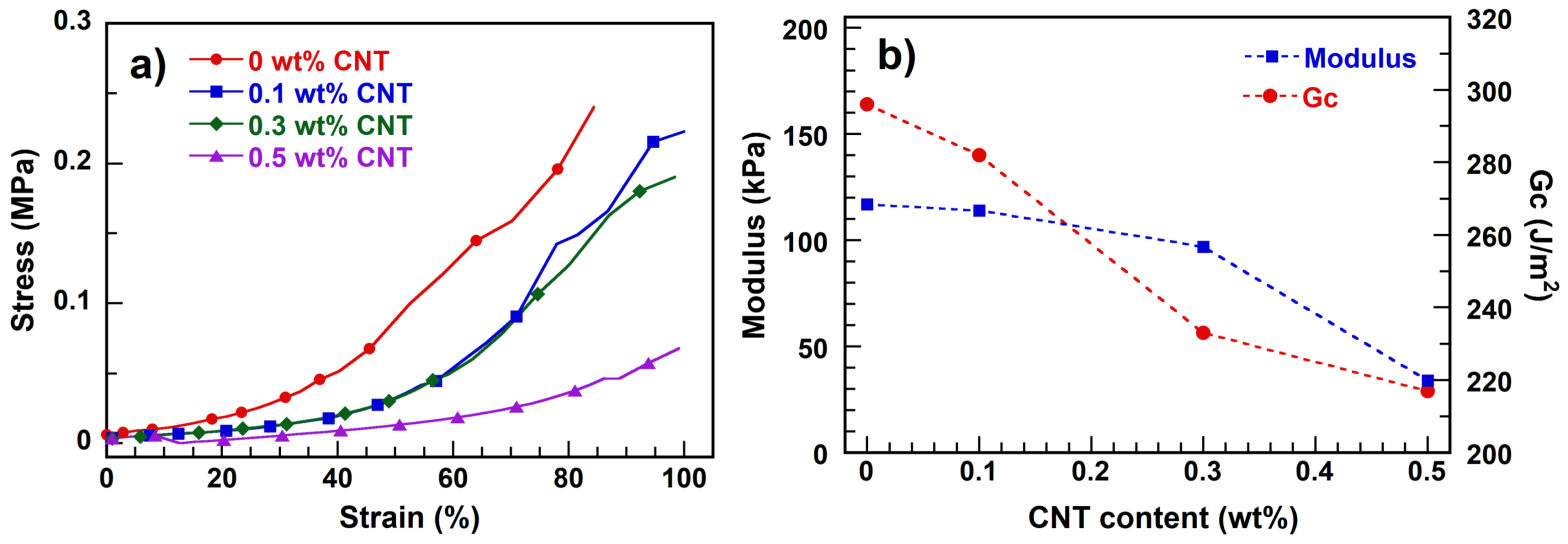
(**a**) Stress-strain curves of self-welding and (**b**) modulus and adhesive fracture energy (*G*_c_) of EPN/CNSL composites with different CNT contents.

T-peeling tests were conducted to examine the self-welding properties of the EPN/CNSL composites. The adhesive fracture energy (*G*_c_) was estimated using Equation (5), as has been reported by Kawashita et al. [51]:

(5) $$G_{c}=\frac{2P\left(1+e\right)}{b}-E_{0}he^{2},$$

where *P* is the average peeling force during steady crack propagation, *E*_0_ is the modulus, *e* is the elastic strain, and *h* and *b* are the thickness and width of the sample, respectively. The stress-strain curves from the T-peeling tests and the values of the modulus and *G*_c_ are given in Figure 8a,b. The highest self-welding performance was observed for EPN/CNSL matrix without CNTs, and the values of *G*_c_ tended to decrease with increasing CNT content. This tendency corresponds to the characteristics of stress relaxation. The presence of CNTs retarded the covalent bond exchange while increasing activation energy (see Figure 7). For self-welding properties, the rate of the BER is a key parameter for the formation of covalent bridges across the contact interface [52]. Capelot et al. [47] reported that the welding strength depends on the concentration of hydroxyl (−OH) groups in epoxy networks, which were controlled by bond exchange via metal-catalyzed transesterification. The *G*_c_ values of EPN/CNSL composites were comparable to those of epoxy vitrimers, e.g., epoxy cured with fatty acid containing 2.24% metal catalyst (232–1032 J/m^2^) [53] and 1% metal catalyst (250−1500 J/m^2^) [54]. According to these results, the self-welding of EPN/CNSL composites without glues or molds would be beneficial for joining or repairing the epoxy networks.

## 4. Conclusions

Semi bio-based EPN/CNSL composites containing 0−0.5 wt % CNT were prepared, and two types of curing processes i.e., etherification and esterification were observed for all of the samples. An increase in CNT content resulted in higher esterification temperatures. After the curing process, an IPN consisting of ester and ether linkage was observed. The presence of 0−0.3 wt % CNTs significantly improved the shape fixities and shape recoveries of the film composites. The shape recovery ratios of the EPN/CNSL composites, under NIR irradiation, showed good recovery, and were nearly 100% over five cycles, indicating that NIR light was absorbed efficiently by the CNTs and converted to thermal energy that was usable for shape recovery. This was supported by the fact that the shape recovery time decreased with increasing CNT content. Furthermore, the reconfigured EPN/CNSL composites displayed shape memory properties. Samples with new shapes can be prepared via transesterification as a BER. The reconfiguration ability and shape memory properties depended on the stress relaxation behaviors. The addition of CNTs suppressed the stress relaxation, resulting in a decrease in adhesive fracture energy. The EPN/CNSL composites can be promising materials that exhibit thermal- and photo-induced shape memory, shape reconfiguration, and self-welding properties. The results of study may provide a new framework for the future design of smart materials.

## Figures and Tables

**Table 1:** Shape memory properties of EPN/CNSL composites activated by NIR light over five cycles.

CNT Content (wt %)	Shape recover ratio (%) at *N* = 1–5					Recovery time (s) at *N* = 1–5				
	1	2	3	4	5	1	2	3	4	5
0	98.85	99.56	99.98	98.89	99.51	230	235	235	230	230
0.1	99.52	98.73	99.67	99.56	99.63	100	90	105	115	120
0.3	98.87	99.13	98.22	99.82	99.56	95	90	85	90	80
0.5	99.45	99.78	98.32	98.67	99.67	65	60	70	65	55

**Table 2:** Shape reconfigure ability and shape memory properties of EPN/CNSL composites activated by NIR light.

CNT Content (wt %)	Original shape	Reconfigured shape	Reconfigured shape at 150 °C	Shape memory behavior activated by NIR	
				Permanent SHAPE	Recovery shape
0	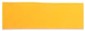	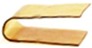	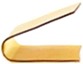	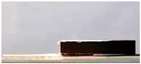	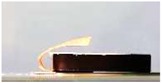
0.1	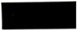	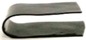	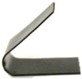	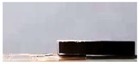	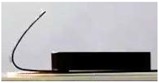
0.3	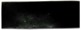	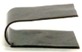	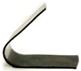	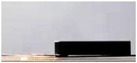	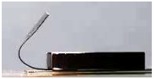
0.5	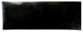	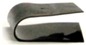	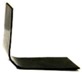	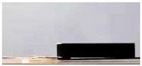	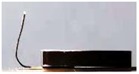

**Table 3:** Shape memory properties of reshaped EPN/CNSL composites activated by NIR light over five cycles.

CNT Content (wt %)	Shape recover ratio (%) at *N* = 1–5					Recovery time (s) at *N* = 1–5				
	1	2	3	4	5	1	2	3	4	5
0	96.35	97.87	88.54	86.33	85.26	390	370	410	500	510
0.1	84.18	77.73	77.53	72.24	71.36	170	210	190	180	190
0.3	79.75	74.47	70.34	67.28	67.48	120	110	130	140	140
0.5	59.73	57.39	59.37	53.43	48.17	80	40	40	40	40

## Supplementary Materials

The following are available online at http://www.mdpi.com/2073-4360/10/5/482/s1, Figure S1: FE-SEM image of CNTs, Figure S2: Preparation of EPN/CNSL composites, Figure S3: DSC curves of EPN/CNSL after curing process, Figure S4: The shape recovery performance test, Figure S5: Configuration of sample for T-peeling test, Figure S6: FT-IR spectra of CNSL, EPN monomer, and EPN/CNSL composites with different CNT contents in the range of (**a**) 800–3500 cm^−1^ and (**b**) 1500–1900 cm^−1^, and (**c**) those of EPN/CNSL matrix in the range of 1550–1900 cm^−1^ at various temperatures, Figure S7: Stress relaxation of EPN/CNSL composites, Table S1: Curing temperature of EPN/CNSL composites, Table S2: Absorption bands in FT-IR spectra and Table S3: Shape memory properties of EPN/CNSL composites activated by NIR light.

## References

[1] Dong Y., Ni Q.-Q., Fu Y. (2015). Preparation and characterization of water-borne epoxy shape memory composites containing silica. Compos. Part A.

[2] Chen Z., Cao R., Ye S., Ge Y., Tu Y., Yang X. (2018). Graphene oxide/poly(*n*-isopropylacrylamide) hybrid film-based near-infrared light-driven bilayer actuators with shape memory effect. Sens. Actuators B Chem..

[3] Lu H., Yao Y., Huang W.M., Leng J., Hui D. (2014). Significantly improving infrared light-induced shape recovery behavior of shape memory polymeric nanocomposite via a synergistic effect of carbon nanotube and boron nitride. Compos. Part B.

[4] Osicka J., Ilčíková M., Mrlik M., Minařík A., Pavlinek V., Mosnáček J. (2017). The impact of polymer grafting from a graphene oxide surface on its compatibility with a pdms matrix and the light-induced actuation of the composites. Polymers.

[5] Ilčíková M., Mrlík M., Sedláček T., Chorvát D., Krupa I., Šlouf M., Koynov K., Mosnáček J. (2014). Viscoelastic and photo-actuation studies of composites based on polystyrene-grafted carbon nanotubes and styrene-*b*-isoprene-*b*-styrene block copolymer. Polymer.

[6] Yi D.H., Yoo H.J., Mahapatra S.S., Kim Y.A., Cho J.W. (2014). The synergistic effect of the combined thin multi-walled carbon nanotubes and reduced graphene oxides on photothermally actuated shape memory polyurethane composites. J. Colloid Interface Sci..

[7] Li H., Zhong J., Meng J., Xian G. (2013). The reinforcement efficiency of carbon nanotubes/shape memory polymer nanocomposites. Compos. Part B.

[8] Song Y., Sun Z., Xu L., Shao Z. (2017). Preparation and characterization of highly aligned carbon nanotubes/ polyacrylonitrile composite nanofibers. Polymers.

[9] Jia X.-L., Zhang Q., Huang J.-Q., Zheng C., Qian W.-Z., Wei F. (2012). The direct dispersion of granular agglomerated carbon nanotubes in bismaleimide by high pressure homogenization for the production of strong composites. Powder Technol..

[10] Montarnal D., Capelot M., Tournilhac F., Leibler L. (2011). Silica-like malleable materials from permanent organic networks. Science.

[11] Brutman J.P., Delgado P.A., Hillmyer M.A. (2014). Polylactide vitrimers. ACS Macro Lett..

[12] Yan P., Zhao W., Fu X., Liu Z., Kong W., Zhou C., Lei J. (2017). Multifunctional polyurethane-vitrimers completely based on transcarbamoylation of carbamates: Thermally-induced dual-shape memory effect and self-welding. RSC Adv..

[13] Huang Z., Wang Y., Zhu J., Yu J., Hu Z. (2018). Surface engineering of nanosilica for vitrimer composites. Compos. Sci. Technol..

[14] Imbernon L., Norvez S., Leibler L. (2016). Stress relaxation and self-adhesion of rubbers with exchangeable links. Macromolecules.

[15] Demongeot A., Mougnier S., Okada S., Soulié-Ziakovic C., Tournilhac F. (2016). Coordination and catalysis of Zn^2+^ in epoxy-based vitrimers. Polym. Chem..

[16] Altuna F., Hoppe C., Williams R. (2016). Shape memory epoxy vitrimers based on dgeba crosslinked with dicarboxylic acids and their blends with citric acid. RSC Adv..

[17] Belmonte A., Russo C., Ambrogi V., Fernández-Francos X., De la Flor S. (2017). Epoxy-based shape-memory actuators obtained via dual-curing of off-stoichiometric “thiol-epoxy” mixtures. Polymers.

[18] Liu W., Fei M.-E., Ban Y., Jia A., Qiu R. (2017). Preparation and evaluation of green composites from microcrystalline cellulose and a soybean-oil derivative. Polymers.

[19] Jahanshahi S., Pizzi A., Abdulkhani A., Shakeri A. (2016). Analysis and testing of bisphenol a-free bio-based tannin epoxy-acrylic adhesives. Polymers.

[20] Nikafshar S., Zabihi O., Moradi Y., Ahmadi M., Amiri S., Naebe M. (2017). Catalyzed synthesis and characterization of a novel lignin-based curing agent for the curing of high-performance epoxy resin. Polymers.

[21] Mathew G., Rhee J., Hwang B., Nah C. (2007). Cure behavior of epoxy resin containing castor oil and cashew nut shell liquid and its derivative. J. Appl. Polym. Sci..

[22] Kasemsiri P., Neramittagapong A., Chindaprasirt P. (2015). Curing kinetic, thermal and adhesive properties of epoxy resin cured with cashew nut shell liquid. Thermochim. Acta.

[23] Wang W., Liu D., Liu Y., Leng J., Bhattacharyya D. (2015). Electrical actuation properties of reduced graphene oxide paper/epoxy-based shape memory composites. Compos. Sci. Technol..

[24] ASTM D1876 (2015) (2015). Standard Test Method for Peel Resistance of Adhesives (T-Peel Test), Astm International, west conshohocken, pa. www.Astm.Org.

[25] Puglia D., Rastin H., Saeb M.R., Shojaei B., Formela K. (2017). Cure kinetics of epoxy/mwcnts nanocomposites: Isothermal calorimetric and rheological analyses. Prog. Org. Coat..

[26] Yang K., Gu M., Jin Y. (2008). Cure behavior and thermal stability analysis of multiwalled carbon nanotube/epoxy resin nanocomposites. J. Appl. Polym. Sci..

[27] Xie H., Liu B., Sun Q., Yuan Z., Shen J., Cheng R. (2005). Cure kinetic study of carbon nanofibers/epoxy composites by isothermal dsc. J. Appl. Polym. Sci..

[28] Mordina B., Tiwari R. (2016). Thermal and mechanical behavior of poly(vinyl butyral)-modified novolac epoxy/multiwalled carbon nanotube nanocomposites. J. Appl. Polym. Sci..

[29] Mwaikambo L., Ansell M. (2001). Cure characteristics of alkali catalysed cashew nut shell liquid-formaldehyde resin. J. Mater. Sci..

[30] Xiong X., Chen P., Ren R., Lu F., Yu Q. (2013). Cure mechanism and thermal properties of the phthalide- containing bismaleimide/epoxy system. Thermochim. Acta.

[31] Kasemsiri P., Hiziroglu S., Rimdusit S. (2011). Effect of cashew nut shell liquid on gelation, cure kinetics, and thermomechanical properties of benzoxazine resin. Thermochim. Acta.

[32] Dušek K. (1986). Network formation in curing of epoxy resins. Epoxy Resins and Composites III.

[33] Ma P.C., Kim J.-K., Tang B.Z. (2007). Effects of silane functionalization on the properties of carbon nanotube/epoxy nanocomposites. Compos. Sci. Technol..

[34] Tang L.-C., Wan Y.-J., Yan D., Pei Y.-B., Zhao L., Li Y.-B., Wu L.-B., Jiang J.-X., Lai G.-Q. (2013). The effect of graphene dispersion on the mechanical properties of graphene/epoxy composites. Carbon.

[35] Tan S.G., Ahmad Z., Chow W.S. (2014). Interpenetrating polymer network structured thermosets prepared from epoxidized soybean oil/diglycidyl ether of bisphenol a. Polym. Int..

[36] Puchot L., Verge P., Peralta S., Habibi Y., Vancaeyzeele C., Vidal F. (2018). Elaboration of bio-epoxy/benzoxazine interpenetrating polymer networks: A composition-to-morphology mapping. Polym. Chem..

[37] Montazeri A., Montazeri N. (2011). Viscoelastic and mechanical properties of multi walled carbon nanotube/epoxy composites with different nanotube content. Mater. Des..

[38] Prasad H.C., Hashmi S., Naik A., Bhargaw H.N. (2017). Improved shape memory effects in multiwalled-carbon-nano-tube reinforced thermosetting polyurethane composites. J. Appl. Polym. Sci..

[39] Leng J., Lan X., Liu Y., Du S. (2011). Shape-memory polymers and their composites: Stimulus methods and applications. Prog. Mater. Sci..

[40] Gu S., Yan B., Liu L., Ren J. (2013). Carbon nanotube–polyurethane shape memory nanocomposites with low trigger temperature. Eur. Polym. J..

[41] Deka H., Karak N., Kalita R.D., Buragohain A.K. (2010). Biocompatible hyperbranched polyurethane/multi-walled carbon nanotube composites as shape memory materials. Carbon.

[42] Ni Q.-Q., Zhang C.-S., Fu Y., Dai G., Kimura T. (2007). Shape memory effect and mechanical properties of carbon nanotube/shape memory polymer nanocomposites. Compos. Struct..

[43] Li G., Meng H. (2015). Recent Advances in Smart Self-Healing Polymers and Composites.

[44] Gardea F., Lagoudas D.C. (2014). Characterization of electrical and thermal properties of carbon nanotube/epoxy composites. Compos. Part B.

[45] Rimdusit S., Lohwerathama M., Hemvichian K., Kasemsiri P., Dueramae I. (2013). Shape memory polymers from benzoxazine-modified epoxy. Smart Mater. Struct..

[46] Altuna F.I., Pettarin V., Williams R.J. (2013). Self-healable polymer networks based on the cross-linking of epoxidised soybean oil by an aqueous citric acid solution. Green Chem..

[47] Capelot M., Montarnal D., Tournilhac F., Leibler L. (2012). Metal-catalyzed transesterification for healing and assembling of thermosets. J. Am. Chem. Soc..

[48] Capelot M., Unterlass M.M., Tournilhac F., Leibler L. (2012). Catalytic control of the vitrimer glass transition. ACS Macro Lett..

[49] Lawton M.I., Tillman K.R., Mohammed H.S., Kuang W., Shipp D.A., Mather P.T. (2016). Anhydride-based reconfigurable shape memory elastomers. ACS Macro Lett..

[50] Ma Z., Wang Y., Zhu J., Yu J., Hu Z. (2017). Bio-based epoxy vitrimers: Reprocessibility, controllable shape memory, and degradability. J. Polym. Sci. A Polym. Chem..

[51] Kawashita L., Moore D., Williams J.G. (2006). Protocols for the measurement of adhesive fracture toughness by peel tests. J. Adhes..

[52] Yu K., Shi Q., Li H., Jabour J., Yang H., Dunn M.L., Wang T., Qi H.J. (2016). Interfacial welding of dynamic covalent network polymers. J. Mech. Phys. Solids.

[53] Shi Q., Yu K., Kuang X., Mu X., Dunn C.K., Dunn M.L., Wang T., Qi H.J. (2017). Recyclable 3D printing of vitrimer epoxy. Mater. Horiz..

[54] Shi Q., Yu K., Dunn M.L., Wang T., Qi H.J. (2016). Solvent assisted pressure-free surface welding and reprocessing of malleable epoxy polymers. Macromolecules.

