# Supplementary material for: Reconfigurable Shape Memory and Self-Welding Properties of Epoxy Phenolic Novolac/Cashew Nut Shell Liquid Composites Reinforced with Carbon Nanotubes
Source: Polymers (Basel). 2018 Apr 28;10(5):482. doi: 10.3390/polym10050482 (PMC6415440; doi:10.3390/polym10050482)
Supplement: Supplementary file 1 [file polymers-10-00482-s001.pdf]

polymers

MDPI

Supplementary Materials

Reconfigurable Shape Memory and Self-Welding Properties of Epoxy Phenolic Novolac/Cashew Nut Shell Liquid Composites Reinforced with Carbon Nanotubes

Pornnapa Kasemsiri [1,*], Narubeth Lorwanishpaisarn [1], Uraiwan Pongsa [2], and Shinji Ando [3,*]

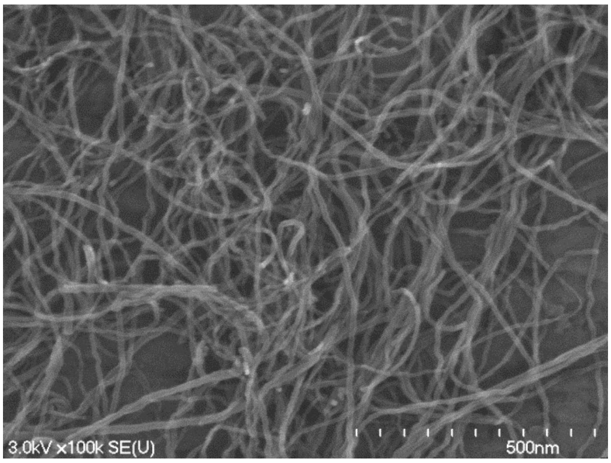

Figure S1. FE-SEM image of CNTs.

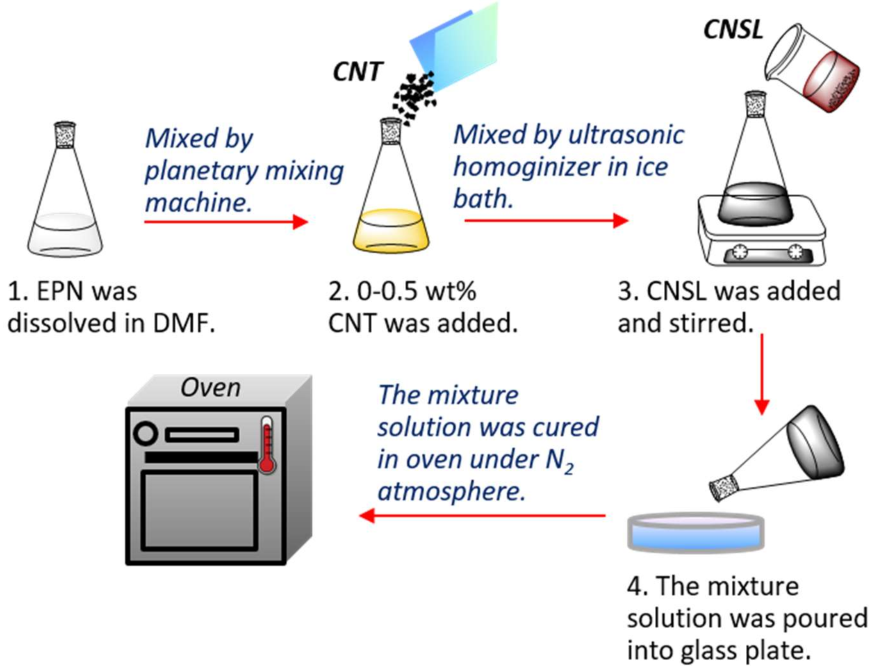

Figure S2. Preparation of EPN/CNSL composites.

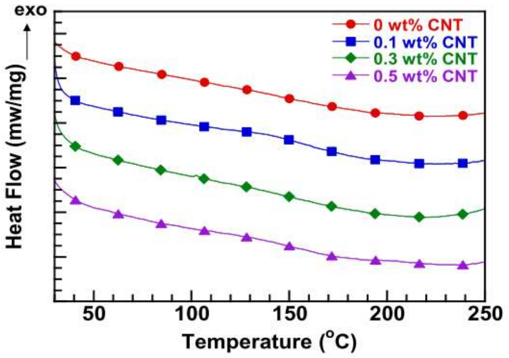

Figure S3. DSC curves of EPN/CNSL after curing process.

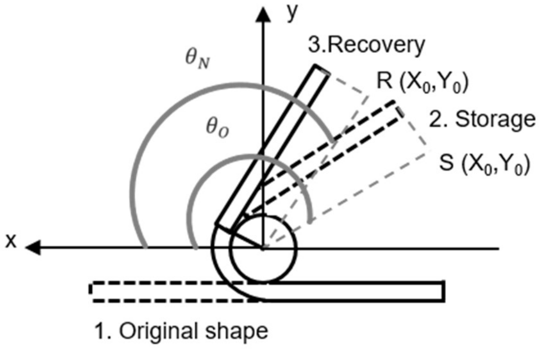

Figure S4. The shape recovery performance test, where, θ_0 was the original storage angle of the specimen in the storage state during the first bending cycle at S (X_0, Y_0). θ_N was the residual angle in the recovery state during the *N*-th thermo-mechanical bending cycle (*N* = 1, 2, 3,...) at $R(X_N, Y_N)$.

$$\text{Recovery ratio}=\frac{\theta_0 - \theta_N}{\theta_0}\times 100\%, \text{ with } \theta_N = \text{Arccot } X_N \quad \text{(S1)}$$

$$(N = 1, 2, 3,..., 0 \leq \theta_N \leq 180°)$$

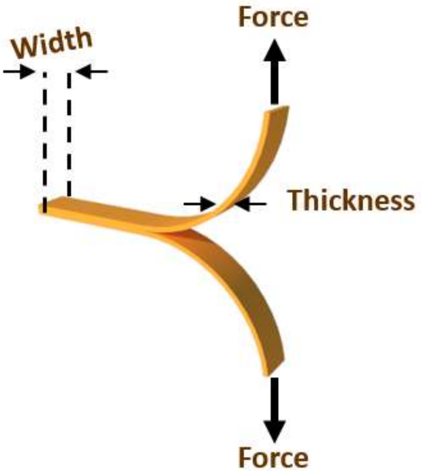

Figure S5. Configuration of sample for T-peeling test.

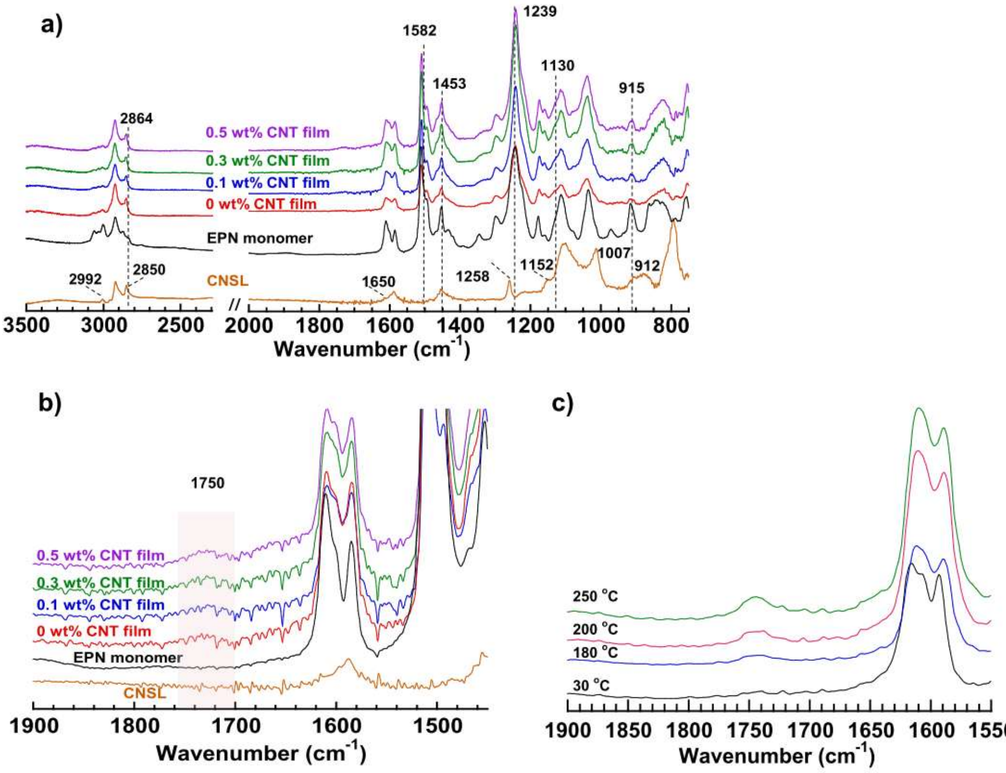

Figure S6. FT-IR spectra of CNSL, EPN monomer, and EPN/CNSL composites with different CNT contents in the range of (**a**) 800–3500 cm^{-1} and (**b**) 1500–1900 cm^{-1}, and (**c**) those of EPN/CNSL matrix in the range of 1550–1900 cm^{-1} at various temperatures.

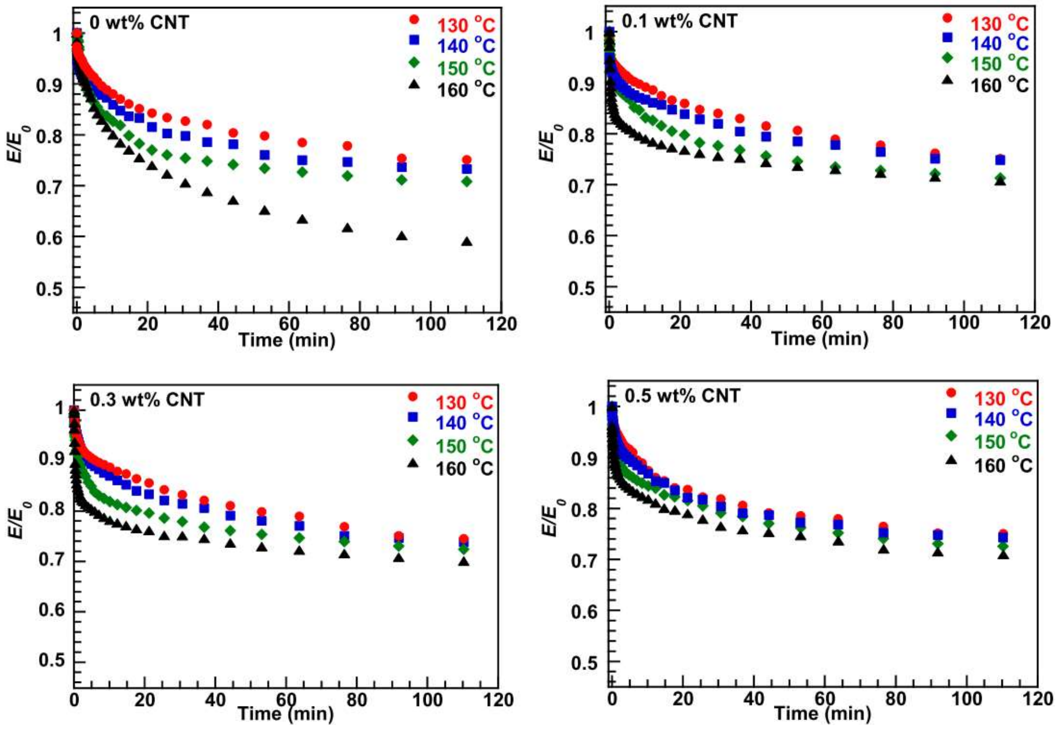

Figure S7. Stress relaxation of EPN/CNSL composites.

Table S1. Curing temperature of EPN/CNSL composites.

CNT content (wt %)	First peak (°C)	Second peak (°C)
0	168	189
0.1	166	193
0.3	166	199
0.5	166	203

Table S2. Absorption bands and their assignments in FT-IR spectra.

Wavenumber (cm^{-1})			**Assignment**
EPN monomer	CNSL	EPN/CNSL film at 0–0.5 wt % CNT	
-	912 and 1007	-	Phenol group at meta position
915	-	915	C–O of epoxide ring
-	-	1130	C–O–C ether linkage
-	1152 and 1258	-	C=C
1239	-	1239	C–O stretching vibration of aromatic
1453	-	1453	CH_2 stretching vibration of epoxide ring
1582	-	1582	C=C stretching vibration of aromatic
-	1650	-	C=O of anarcardic acid
-	-	1750	C=O–O–C ester linkage
	2850 and 2992		CH_3 stretching
2864		2864	CH stretching

Table S3. Shape memory properties of EPN/CNSL composites activated by NIR light.

**CNT content (wt %)**	**Original shape**	**Shape memory behavior activated by NIR**	
		**Permanent shape**	**Recovery shape**
**0**	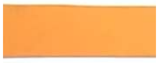	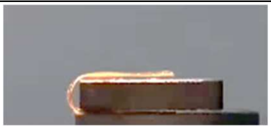	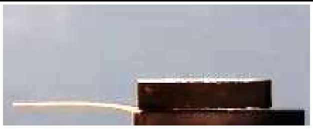
**0.1**	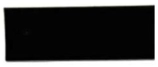	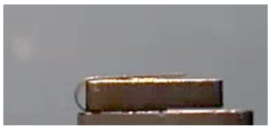	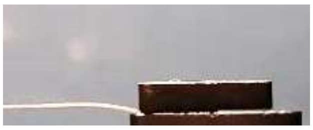
**0.3**	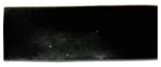	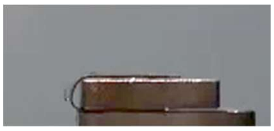	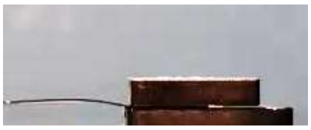
**0.5**	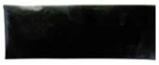	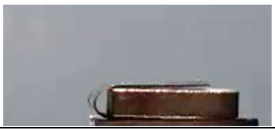	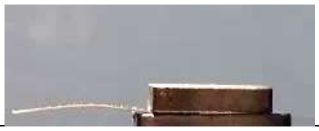

© 2018 by the authors. Submitted for possible open access publication under the terms and conditions of the Creative Commons Attribution (CC BY) license (http://creativecommons.org/licenses/by/4.0/).
